# MHD mixed convection and entropy generation of CNT-water nanofluid in a wavy lid-driven porous enclosure at different boundary conditions

**DOI:** 10.1038/s41598-022-06957-3

**Published:** 2022-02-21

**Authors:** Hameed K. Hamzah, Farooq H. Ali, M. Hatami

**Affiliations:** 1 Mechanical Engineering Department, College of Engineering, University of Babylon, Babylon, Iraq; 2 Department of Mechanical Engineering, Ferdowsi University of Mashhad, Mashhad, Iran

**Keywords:** Fluid dynamics, Computational nanotechnology, Computational science

## Abstract

In this study, Galerkin Finite Element Method or GFEM is used for the modeling of mixed convection with the entropy generation in wavy lid-driven porous enclosure filled by the CNT-water nanofluid under the magnetic field. Two different cases of boundary conditions for hot and cold walls are considered to study the fluid flow (streamlines) and heat transfer (local and average Nusselt numbers) as well as the entropy generation parameters. Richardson (Ri), Darcy (Da), Hartmann angle (γ), Amplitude (A), Number of peaks (N), Volume fraction (φ), Heat generation factor (λ), Hartmann number (Ha) and Reynolds number (Re) are studied parameters in this study which results indicated that at low Richardson numbers (< 1) increasing the inclined angle of magnetic field, decreases the Nu numbers, but at larger Richardson numbers (> 1) it improves the Nu numbers.

## Introduction

The rapid development of electronic devices, solar collectors, heating elements, heat exchangers, drying and lubricant techniques, it pushes us to find an effective way to increase the efficiency of heat transfer effectively and to reach that effectiveness it became necessary to use the combined method (passive-active). Using nanofluid and porous medium is an active method while the wall modification with sinusoidal style is the passive method. Lots industrial applications that depend on its work of lid-driven cavity such as chemical etching, film coating, industrial drying process, industrial coating, short dwell coaters utilizing for the manufacturing of high quality photographic films and papers, roll coating, several color printings, polymer processing apparatus design, Bingham plastics flow, dryers and solar collectors. Free and forced convection of heat transfer from nanofluids such as CNT-water has many applications such as cooling processes in industries which motivated the researchers to work on this phenomena^1^. Furthermore, different ways and techniques such as magnetic field applying and porous media usage is used to improve or control the heat transfer amount in those applications^1,2^. Hamida and Hatami^3^ used the electrical field in their square light emitting diode modeling to improve the cooling process from microchannel filled by nanofluid. Ghasemi et al.^4^ and Hamzah et al.^5^ used the magnetic field to improve the heat transfer in the solar radiation application and immersed rotating cylinder, respectively. Behzadnia et al.^6,7^ modeled the super-critical nanofluid flow for improving the cooling process in reactors and Hatami^8^, and Hatami and Safari^9^ improved the nanofluid heat transfer from cavities by using heated fins and cylinders, respectively. Recently, Nakhchi et al.^10^ investigated the CuO-water heat transfer from heat exchanger using the novel perforated turbulators as external devices.

Many researchers tried to improve the heat transfer of nanofluids using new technologies. Shaker et al.^11^ used the non-uniform magnetic field for the mixed convection of open cavity and found maximum 57.07% Nu improvements by magnetic number. Wang et al.^12^ used the porous twisted tapes for heat transfer improvement of silica-water (SiO_2_–H_2_O) nanofluids in rounds and triangular tubes. Al-Farhany and Abdulsahib^13^ considered the mixed convection of nanofluid in porous medium over a rotating cylinder and found the higher Darcy number and clockwise rotation causes a maximum Nusselt number. Nong et al.^14^ used the control volume finite element (CVFE) method to study the Lorentz force effect on the free convection of a wavy cavity and concluded that nanoparticles shape insignificantly influences the average Nusselt number. Abbas et al.^15^ used magnetic field and Ag/Ni-water hybrid nanofluids to improve the heat transfer over a cylinder, analytically. Electrical field applying to the nanofluids heat trasnfer is another technique which recently is used by researchers such as Chen et al.^16^, numerically. They concluded that the electric field forces modify the velocity field and temperature.

Among the studies on the external fields, magnetic field is more used by researchers than an electric field due to its more effect on the nanoparticles. Recently, Aly et al.^17^ investigated the effect of magnetic field on the finned cavity, including a rotating rectangle and reported that mean rates of heat and mass transfer decreased by increasing the Hartmann number. Berrahil et al.^18^ applied the magnetic field on the Al_2_O_3_/water, natural convection in an annular enclosure and found that the rate of the average Nu number, decrement caused by the magnetic field, is greater as the radius ratio λ decreases. Mourad et al.^19^ used the uniform magnetic field for the thermal performance of a wavy cavity filled by Fe_3_O_4_-MWCNT hybrid nanofluid and confirmed that Nu improves by Darcy and porosity number, while it reduced by Ha number. Alsabery et al.^20^ used at the same time magnetic field and rotating cylinders in a wavy surface and stated that rotation of the cylinders can enhance the Nu number up to 315%. Also, Zhang and Zhang^21^ studied the effect of magnetic field direction on the heat transfer of Fe_3_O_4_-water nanofluid, numerically. They described that 8% increase in convective heat transfer is observed when the magnetic direction was perpendicular to the flow direction. Also, in another study, they^22^ confirmed that the convective heat transfer coefficient increases with the increase of alternating frequency for the magnetic field, experimentally. Not only the magnetic field is used for heat transfer of nanofluids in cavities^23^, but also porous media are considered as a wavy of heat transfer improvement as seen in the literature^24^. Sara et al.^25^ in this review study deals with natural convection in multi-shapes cavities and effective parameters such as magnetic field, nanoparticle, porosity, obstruction body and inclination angle of the cavity. Mohammad et al.^26^ the aim of this work focused an enhance heat transfer in the helical tube heat exchanger having non-Newtonian nanofluid taking into account the following factors such as cost, dimensions, and thermal system energy storage. The result shows that maximum performance can reduce to 28%. Hamid et al.^27^ in the current study, an inclusive review was made of the utilized of nanofluid in heat pipe thermosyphon. The execution of thermosyphon relies on many factors such as types of nanofluid and its concentration, using of surfactant and the amount of heat supplied. Also the study deals with of nanofluid thermosyphon implementation is systems of energy. Ahmed et al.^28^ in the current work a very valuable historical review that deals with nanofluids, types of nanomaterial, base fluid and surfactant. Application in different energy systems, advantage and disadvantage in a very detailed and accurate manner. Sara et al.^29^ the study deals with the influence of using sound waves of measuring viscosity of ethylene glycol with SiO_2_ nanofluid. Many factors were student like temperature, mass fraction, rate of shear and time of a sound wave and its effect on viscosity by two method step. Quyan et al.^30–33^ in this works, a numerical investigation was used to simulate heat transfer, fluid flow and entropy generation in three different channels, the first one is double pipe wavy wall heat exchanger, the second one is corrugated wall with triangular shape and the third is a microchannel injection from top wall. The working fluid, a novel admixture of FMWNT-water (Functionilized Multi-Walled Carbon Nano-Tubes) based water is exercised as the working fluid. The flow is subjected to magnetic strength with constant value. The results show that using magnetic flux with this type of nanofluid was effective in improving heat transfer and reducing the entropy generation. Masoud et al.^34^ during this search, controlling heat transfer by applying the magnetic field technique to the conductive electrical fluid (melting gallium) in circular enclosure was displayed. Many parameters are studied, magnetic field angle, Rayleigh number, magnetic strength and inner to outer radius ratio, The results indicated that increasing magnetic field angle, Rayleigh and radius ratio lead to increase the heat transfer Nusselt number. Ehsan et al.^35^ calculated the thermal conductivity and viscosity of FMWCN (Functionalized Multi-Walled Carbon Nanotubes) from experimental results and using this properties to simulate heat transfer and fluid flow of non-Newtonian fluid in circular pipe and constant heat flux. Results indicate that this type of nanoparticles was more convenient with great shear fluid rate. Liang et el.^36^ the aim of this study is to make a comparison between the common rectangular channel and corrugated one in the presence of discrete heat sources in the upper straight surface. The results display that when the discrete heat source near the peak of undulation surface has more effective than other cases.

Abanoub et al.^37^ in the recent current study, the problem of lid-driven of two dimensional incompressible flow using a finite difference technique. In this work, most of industrial applications in which it is possible to be noticed the phenomenon of mixed convection by lid-driven.

So, in this study, it is trying to do a complete study on the mixed convection heat transfer of nanofluids under the magnetic field in a porous medium which has a movable wavy wall with different boundary conditions, numerically. The study goes for the first time that dealing with lid-drive having wavy in shape and movement with presence of many factors such as CNT-nanomaterial, porous medium, heat generation, effect of magnetic field and its angle.

## Mathematical description of the problem

**Figure 1:**
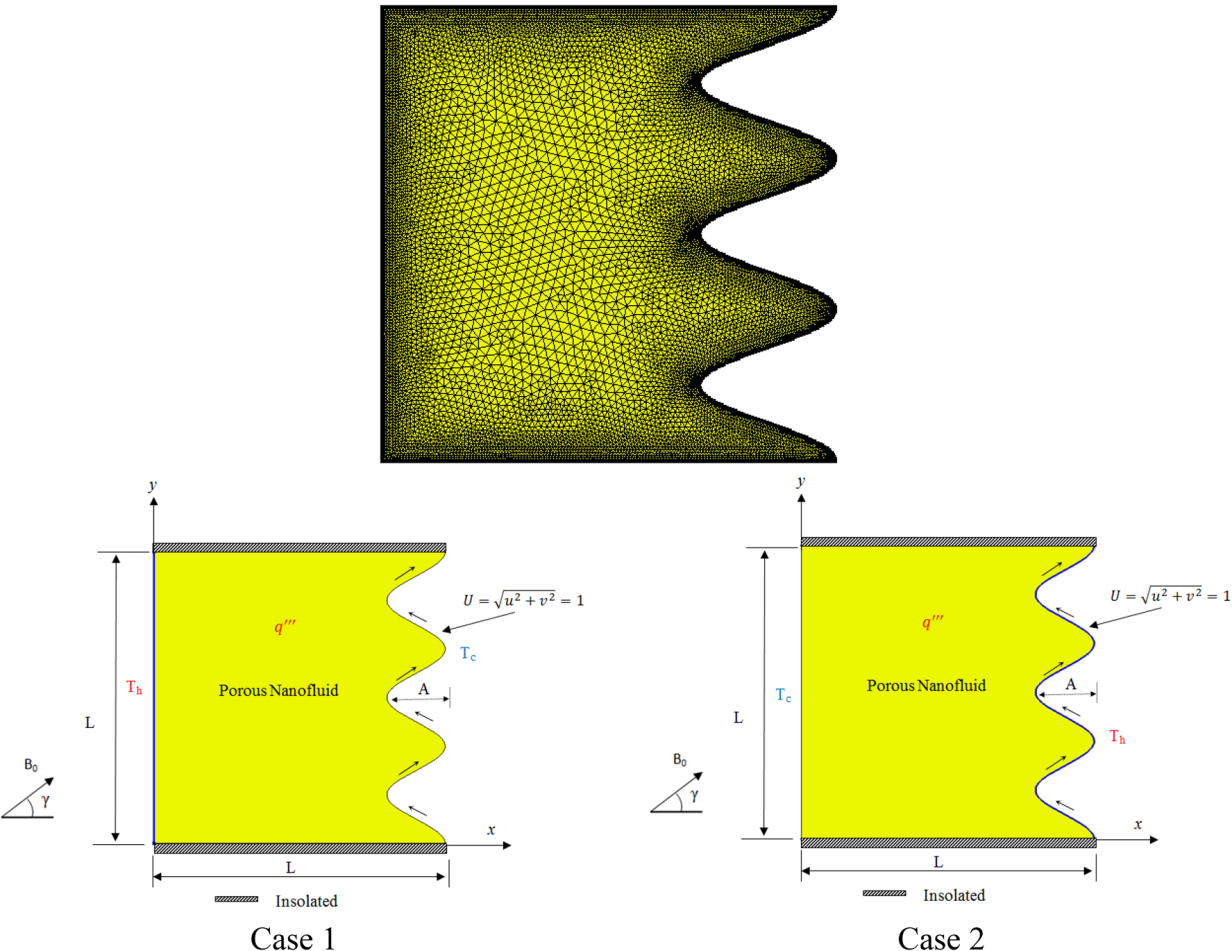
Mesh generation and different boundary conditions in two cases.

The problem geometry is schematically explained in Fig. 1, it is two dimensional porous cavity of similar length and height equal to unity filled by CNT-nanofluid. CNT are non-Newtonian fluids, whose high viscosity obstructs convection and leads to acceptable heat transfer coefficient under mixed convection, despite their high thermal conductivity. The upper and lower boundaries are insulated where the straight right and wavy left walls are in cold and hot temperatures in two different cases. The right wall is wavy with a number of peaks (N) and amplitude (A), this wavy wall is moved to upward with the velocity of U = 1. A magnetic field which strength of B_0_ and angle of γ is applied to the cavity. Uniform heat generation (qʺ) was applied entire the cavity. The assumptions of this model are incompressible, steady state, laminar flow, homogenous isotropic porous medium, single phase nanofluid. The model of porous media is a Darcy Brinkman model. The parameters neglected are radiation effect and viscous dissipation. Under the above assumptions, the conservation of mass, and in the case of mixed convection, and also the conservation of momentum energy equations can be written as^2^:

1 $$ \nabla u = 0 $$

2 $$ \frac{{\rho_{nf} }}{{\varepsilon^{2} }}u.\nabla u = - \nabla p - \frac{{\mu_{nf} }}{K}u + \frac{{\mu_{nf} }}{\varepsilon }\nabla^{2} u + \rho_{nf} \beta_{nf} \left( {T - T_{0} } \right)g + J \times B^{*} $$

3 $$ u.\nabla T = \alpha_{eff,nf} \nabla^{2} T + \frac{{Q_{0} }}{{\left( {\rho C_{p} } \right)_{nf} }} $$

4 $$ \nabla^{2} \Omega = B^{*} .\nabla \times u $$

5 $$ J = \sigma_{nf} \left( { - \nabla \Omega + u \times B^{*} } \right) $$

where ($\nabla u$) shows the velocity vector and K denotes the permeability of porous media, J is electrical current and B^*^ refers to external magnetic field. Ω is the electric potential, and σ_nf_ is the electrical conductivity. More information on the parameters can be found in^2^. Chamkha et al.^2^ showed that the expressed equations are:

6 $$ \frac{\partial u}{{\partial x}} + \frac{\partial v}{{\partial y}} = 0 $$

7 $$ \frac{1}{{\varepsilon^{2} }}\left( {u\frac{\partial u}{{\partial x}} + v\frac{\partial u}{{\partial y}}} \right) = - \frac{1}{{\rho_{nf} }}\frac{\partial p}{{\partial x}} + \frac{{\upsilon_{nf} }}{\varepsilon }\left( {\frac{{\partial^{2} u}}{{\partial x^{2} }} + \frac{{\partial^{2} u}}{{\partial y^{2} }}} \right) - \frac{{\upsilon_{nf} }}{K}u + \frac{{\sigma_{nf} B_{0}^{2} }}{{\rho_{nf} }}\left( {v\sin \gamma \cos \gamma - u\sin^{2} \gamma } \right) $$

8 $$ \frac{1}{{\varepsilon^{2} }}\left( {u\frac{\partial v}{{\partial x}} + v\frac{\partial v}{{\partial y}}} \right) = - \frac{1}{{\rho_{nf} }}\frac{\partial p}{{\partial y}} + \frac{{\upsilon_{nf} }}{\varepsilon }\left( {\frac{{\partial^{2} v}}{{\partial x^{2} }} + \frac{{\partial^{2} v}}{{\partial y^{2} }}} \right) - \frac{{\upsilon_{nf} }}{K}v + \left( {\frac{{\left( {\rho \beta } \right)_{nf} }}{{\left( \rho \right)_{nf} }}} \right)g(T - T_{0} ) + \frac{{\sigma_{nf} B_{0}^{2} }}{{\rho_{nf} }}\left( {v\sin \gamma \cos \gamma - u\cos^{2} \gamma } \right) $$

9 $$ u\frac{\partial T}{{\partial x}} + v\frac{\partial T}{{\partial y}} = \alpha_{eff,nf} \left( {\frac{{\partial^{2} T}}{{\partial x^{2} }} + \frac{{\partial^{2} T}}{{\partial y^{2} }}} \right) + \frac{{Q_{0} }}{{\left( {\rho c_{p} } \right)_{nf} }}(T - T_{0} ) $$

Also, they showed that by considering the following non-dimentional parameters:

10 $$X, Y=\frac{x, y}{L}; U, V=\frac{(u, v)}{{V}_{o}}; \theta =\frac{(T-{T}_{o})}{\Delta T}; {T}_{o}=\frac{{T}_{h}-{T}_{c}}{2};Ri=\frac{Gr}{{Re}^{2}};Q=\frac{{Q}_{o}{L}^{2}}{{\left(\rho {c}_{p}\right)}_{f}};P=\frac{p}{{\rho }_{nf}{V}_{o}^{2}}; {S}_{l}={S}_{r}=\frac{S{\mu }_{f}}{L}$$

The non-dimentional form of governing equation will be in the form of:

11 $$ \frac{\partial U}{{\partial X}} + \frac{\partial V}{{\partial Y}} = 0 $$

12 $$ \frac{1}{{\varepsilon^{2} }}\left( {U\frac{\partial U}{{\partial X}} + V\frac{\partial U}{{\partial Y}}} \right) = - \frac{\partial P}{{\partial X}} + \frac{1}{{\varepsilon {\text{Re}} }}\frac{{\rho_{f} \mu_{nf} }}{{\rho_{nf} \mu_{f} }}\left( {\frac{{\partial^{2} U}}{{\partial X^{2} }} + \frac{{\partial^{2} U}}{{\partial Y^{2} }} - \frac{\varepsilon U}{{Da}}} \right) + \frac{{Ha^{2} }}{{\text{Re}}}\left( {\frac{{\rho_{f} }}{{\rho_{nf} }}} \right)\left( {\frac{{\sigma_{nf} }}{{\sigma_{f} }}} \right)\left( {V\sin \gamma \cos \gamma - U\sin^{2} \gamma } \right) $$

13 $$  \frac{1}{{\varepsilon^{2} }}\left( {U\frac{\partial V}{{\partial X}} + V\frac{\partial V}{{\partial Y}}} \right)  = - \frac{\partial P}{{\partial Y}} + \frac{1}{{{\text{Re}} \varepsilon }}\frac{{\rho_{f} \mu_{nf} }}{{\rho_{nf} \mu_{f} }}\left( {\frac{{\partial^{2} V}}{{\partial X^{2} }} + \frac{{\partial^{2} V}}{{\partial Y^{2} }} - \frac{\varepsilon V}{{Da}}} \right) + \left( {\frac{{\left( {\rho \beta } \right)_{nf} }}{{\left( {\rho \beta } \right)_{f} }}} \right)Ri\theta + \frac{{Ha^{2} }}{{\text{Re}}}\left( {\frac{{\rho_{f} }}{{\rho_{nf} }}} \right)\left( {\frac{{\sigma_{nf} }}{{\sigma_{f} }}} \right)\left( {U\sin \gamma \cos \gamma - V\cos^{2} \gamma } \right)  $$

14 $$ U\frac{\partial \theta }{{\partial X}} + V\frac{\partial \theta }{{\partial Y}} = \frac{1}{{\Pr {\text{Re}} }}\frac{{\alpha_{nf} }}{{\alpha_{f} }}\left( {\frac{{\partial^{2} \theta }}{{\partial X^{2} }} + \frac{{\partial^{2} \theta }}{{\partial Y^{2} }}} \right) + \frac{1}{{\Pr {\text{Re}} }}\frac{{\left( {\rho c_{p} } \right)_{f} }}{{\left( {\rho c_{p} } \right)_{nf} }}Q\theta $$

where

15 $$Pr=\frac{{\nu }_{f}}{{\alpha }_{eff.f}};\, Re=\frac{{V}_{o}L}{{\nu }_{f}};\, Gr=\frac{g{\beta }_{f}{L}^{3}\Delta T}{{\nu }_{f}^{2}};\, Ha={B}_{o}L\sqrt{\frac{\sigma_{f}}{\mu _{f}}};\, Da=\frac{K}{{L}^{2}};\, {\alpha }_{eff.nf}=\frac{{k}_{eff.nf}}{{\left(\rho {c}_{p}\right)}_{nf}};\,  {\alpha }_{eff.f}=\frac{{k}_{eff.f}}{{\left(\rho {c}_{p}\right)}_{f}}$$

The local Nusselt number will be

16 $$ Nu_{s} = - \frac{{k_{eff,nf} }}{{k_{eff,f} }}\left( {\frac{\partial \theta }{{\partial Y}}} \right) $$

and the non-dimensional entropy generation, (S), can be written as^2^

17 $$ \begin{aligned} S & = s \cdot \frac{{H^{2} \cdot T_{0}^{2} }}{{k_{f} \left( {\Delta T} \right)^{2} }} = \left( {\frac{{k_{eff,nf} }}{{k_{eff,f} }}} \right)\left[ {\left( {\frac{\partial \theta }{{\partial X}}} \right)^{2} + \left( {\frac{\partial \theta }{{\partial Y}}} \right)^{2} } \right] \\& \quad + \Theta \cdot \left( {\frac{{\mu_{nf} }}{{\mu_{f} }}} \right) \cdot {\text{Re}}^{2} \cdot \Pr^{2} \left\{ {\frac{1}{Da}(U^{2} + V^{2} ) + 2\left[ {\left( {\frac{\partial U}{{\partial X}}} \right)^{2} + \left( {\frac{\partial V}{{\partial Y}}} \right)^{2} } \right] + \left( {\frac{\partial V}{{\partial X}} + \frac{\partial U}{{\partial Y}}} \right)^{2} } \right\} \\ & \quad + \Theta \cdot \left( {\frac{{\sigma_{nf} }}{{\sigma_{f} }}} \right) \cdot {\text{Ha}}^{2} \cdot {\text{Re}}^{2} \cdot \Pr^{2} \cdot (U\sin \Phi - V\cos \Phi )^{2} = S_{h} + S_{v} + S_{j} , \\ \end{aligned} $$

where S_h_, S_v_, and S_j_ are the dimensionless local entropy generation rate due to heat transfer, the fluid fraction, and the Joule heating, respectively. Θ is the irreversibility factor which represents the ratio of the viscous entropy generation to thermal entropy generation

18 $$ \Theta = \frac{{\mu_{f} T_{0} }}{{k_{f} }}\left( {\frac{{\alpha_{eff,f} }}{\Delta T.H}} \right)^{2} $$

The Bejan number, Be, defined as the ratio between the entropy generation due to heat transfer by the total entropy generation:

19 $$ Be = \frac{Sh}{S} $$

More details of defined parameters such as average Nusselt number, boundary conditions, and nanofluid properties in the governing equations can be found in^2^.

The density, heat capacity, thermal expansion, thermal diffusivity, thermal conductivity, viscosity and the electrical conductivity are explained by the following equations:

20 $${\rho }_{nf}=\left(1-\varphi \right){\rho }_{f}+\varphi {\rho }_{sp}$$

21 $${\left(\rho {c}_{p}\right)}_{nf}=\left(1-\varphi \right){\left(\rho {c}_{p}\right)}_{f}+\varphi {\left({\rho c}_{p}\right)}_{sp}$$

22 $${\left(\rho \beta \right)}_{nf}=\left(1-\varphi \right){\left(\rho \beta \right)}_{f}+\varphi {\left(\rho \beta \right)}_{sp}$$

23 $${\alpha }_{nf}=\frac{{k}_{nf}}{{\left(\rho {c}_{p}\right)}_{nf}}$$

24 $$\frac{{k}_{nf}}{{k}_{f}}=\frac{\left({k}_{sp}+2{k}_{f}\right)-2\varphi \left({k}_{f}-{k}_{sp}\right)}{\left({k}_{sp}+2{k}_{f}\right)+\varphi \left({k}_{f}-{k}_{sp}\right)}$$

25 $${k}_{eff, nf}=\varepsilon {k}_{nf}+\left(1-\varepsilon \right){k}_{sp}$$

26 $${k}_{eff, f}=\varepsilon {k}_{f}+\left(1-\varepsilon \right){k}_{sp}$$

27 $${\alpha }_{eff, nf}=\frac{{k}_{eff,nf}}{{\left(\rho {c}_{p}\right)}_{nf}}$$

28 $${\mu }_{nf}=\frac{{\mu }_{f}}{{\left(1-\varphi \right)}^{2.5}}$$

29 $$\frac{{\sigma }_{nf}}{{\sigma }_{f}}=1+\frac{3\left(\delta -1\right)\varphi }{\left(\delta +2\right)-\left(\delta -1\right)\varphi }$$

where $$\delta =\frac{{\sigma }_{nf}}{{\sigma }_{f}}$$.

## Numerical method

In this study, Galerkin weighted residual FEM (GFEM) is used for the explanation of the governing equations and the boundary conditions by the software package COMSOL Multiphysics 5.6 [https://www.comsol.com/release/5.6]. At the beginning, thus named Galerkin weighted residual is progressed by conquest appropriate grid number of elements ad a gradation of lattices is intended from the rough grid at G1. The next grid of next mesh is repeated for smooth grid G2 by rising number of elements as can noticed in Table 2. So, the full domain is divided into non-mapping elements ${\Omega }_{R}$, $R\in N$ at every mesh grid. So as to realize the shape function on every element ${\Omega }_{R}$, a domestic sign coordinate system (ξ, η) is inserted. Here, the nanofluid considered single-phase and incompressible, by laminar flow (spf) module and the heat transfer in the media (ht) are used for the modeling of Eqs. (11)–(14) with nanofluid properties of Table 1. To model the magnetic field, the source terms of the governing equations have changed in the software in this demonstrating. Additionally, to state the stability of the solution, Galerkin least-squares method and P2-P1 Lagrange elements were used. In the present numerical solution, the convergence criteria are defined by the error estimation setting to $\left| {M^{m + 1} - M^{m} } \right| \le 10^{ - 5}$, where *m* shows the iteration number during the solution and *M* refers to $(u,v,T,V)$ parameters as the general dependent variables.

**Table 1:** CNTs thermal properties^1^.

Properties	C_p_(J/kg k)	ρ (kg/m^3^)	k (W/m k)	µ (kg/m s)	$\sigma$(S/m)
SWCNT	425	2600	6600	–	2.54e4
MWCNT	796	1600	3000	–	4.95e3
Pure water	4179	997.1	0.613	0.000889	0.05

## Results and discussion

**Figure 2:**
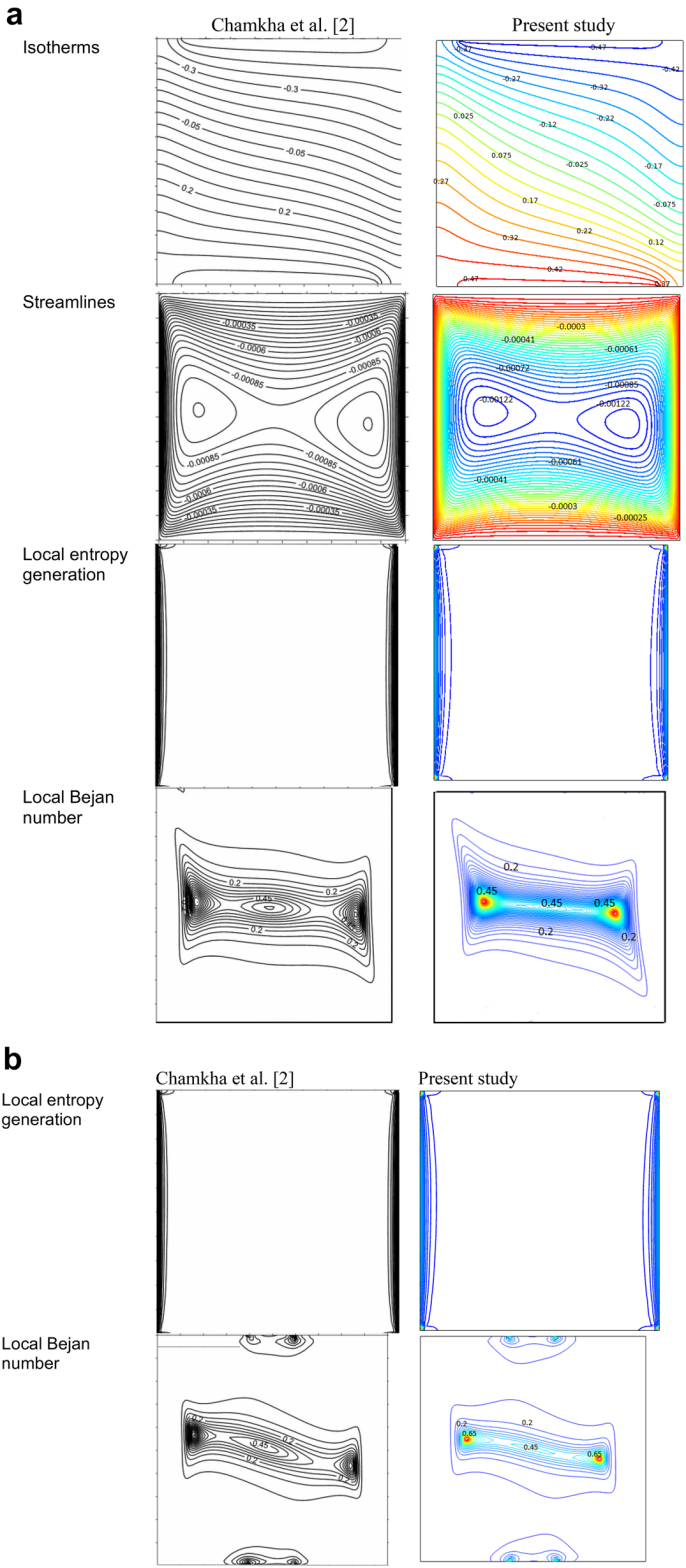
(**a**) Comparison of the present study with *Re* = 100, *Pr* = 6.2, *Ri* = 1, φ = 0.05, *Ha* = *10, γ* = 45, Da = 1e−3, Q = 1.B = 0.8, D = 0.5. (**b**) Comparison of the present study with *Re* = 100, *Pr* = 6.2, *Ri* = 1, φ = 0.05, *Ha* = *10, γ* = 45, Da = 1e−3, Q = 1.B = 0.2, D = 0.5.

As discussed above, in this manuscript the effect of mixed convection of CNT-water nanofluid in a wavy porous cavity is investigated under the magnetic field. As shown in Fig. 1, the wavy wall is moved by a unit velocity and two cases are considered for the temperature boundary layers. In case 1 the straight left hand side wall is at high temperature (T_h_) while the wavy right hand side is a low temperature (T_c_). For Case 2, the T_h_ and T_c_ temperatures are replaced as shown in Fig. 1. Table 1 shows the CNTs thermal properties obtained from^1^. To reach a grid independence study, Table 2 is studied for different grid numbers. Five different grid numbers are tested and results of an average Nusselt number, time of the solution and ψ_min_ were presented. As seen, G5 and G6 elements have excellent results which are more close to each other, but G5 due to lower time of solution can be chosen as the suitable grind number for this study. To have a validation study, the results of the present numerical method are compared with those of Chamkha et al.^2^ in a square cavity as shown in Fig. 2a,b. As seen, the current results have excellent agreement with those of Chamkha et al.^2^ for Isotherms, Streamlines, Entropy generations and Bejan numbers contours. In the next steps, effects of different parameters which are presented by their values in Table 3, are investigated on the results. Richardson (Ri), Darcy (Da), Hartmann angle (γ), Amplitude (A), Number of peaks (N), Volume fraction (φ), Heat generation factor (λ), Hartmann number (Ha) and Reynolds number (Re) are the considered parameters in this study. Following the results of the two cases are presented.

**Table 2:** Grid independent test average Nusselt number on hot surface at (Re = 200, Ri = 10, Da = 1e−3 λ = 5, φ = 0.04, Ha = 20, γ = 45, A = 0.15, N = 3).

Grid	Domain elements	Boundary elements	Time (sec)	Nu_ave_	$\left|Error\right|\%$	$\left|{\psi }_{min}\right|$	Error $\%$
G1	3259	247	27	13.173	–	0.034794	–
G2	4917	307	29	13.181	0.06	0.034796	0.0057
G3	8521	453	36	13.154	0.205	0.034795	0.0028
G4	**19,721**	**797**	**46**	**13.144**	**0.076**	**0.034826**	**0.089**
G5	26,527	797	70	13.144	0	0.034838	0.0344

Significant values are in bold.

**Table 3:** Parameters under study.

Ri	Richardson number	0.01,0.1,1,10
Da	Darcy number	1e−1,1e−2, 1e3,1e4,1e−5
γ	Hartman angle	0,30,45,60,90
A	Amplitude	0.05 0.15 0.25
N	Number of peaks	1 2 3 4 5
φ	Volume fraction	0,0.02, 0.04, 0.06
λ	Heat generation factor	−5 0 5
Ha	Hartman number	0–40
Re	Reynolds number	100–500

### Case 1

**Figure 3:**
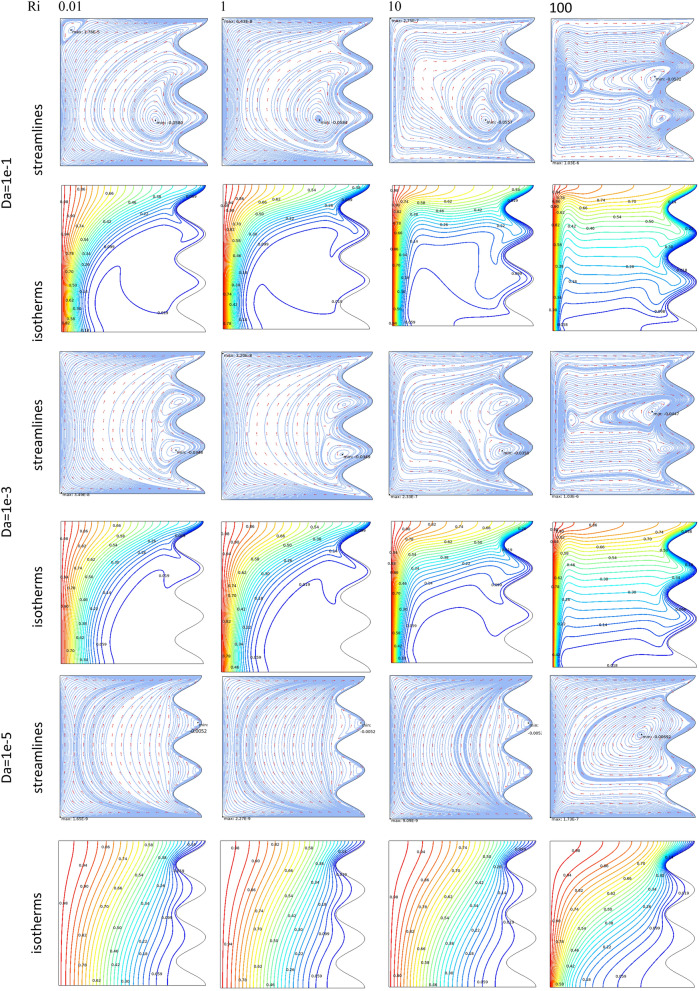
Streamlines and isotherms at (Re = 100, λ = 5, φ = 0.02, Ha = 25, γ = 45, A = 0.1, N = 3) for Case 1.

**Figure 4:**
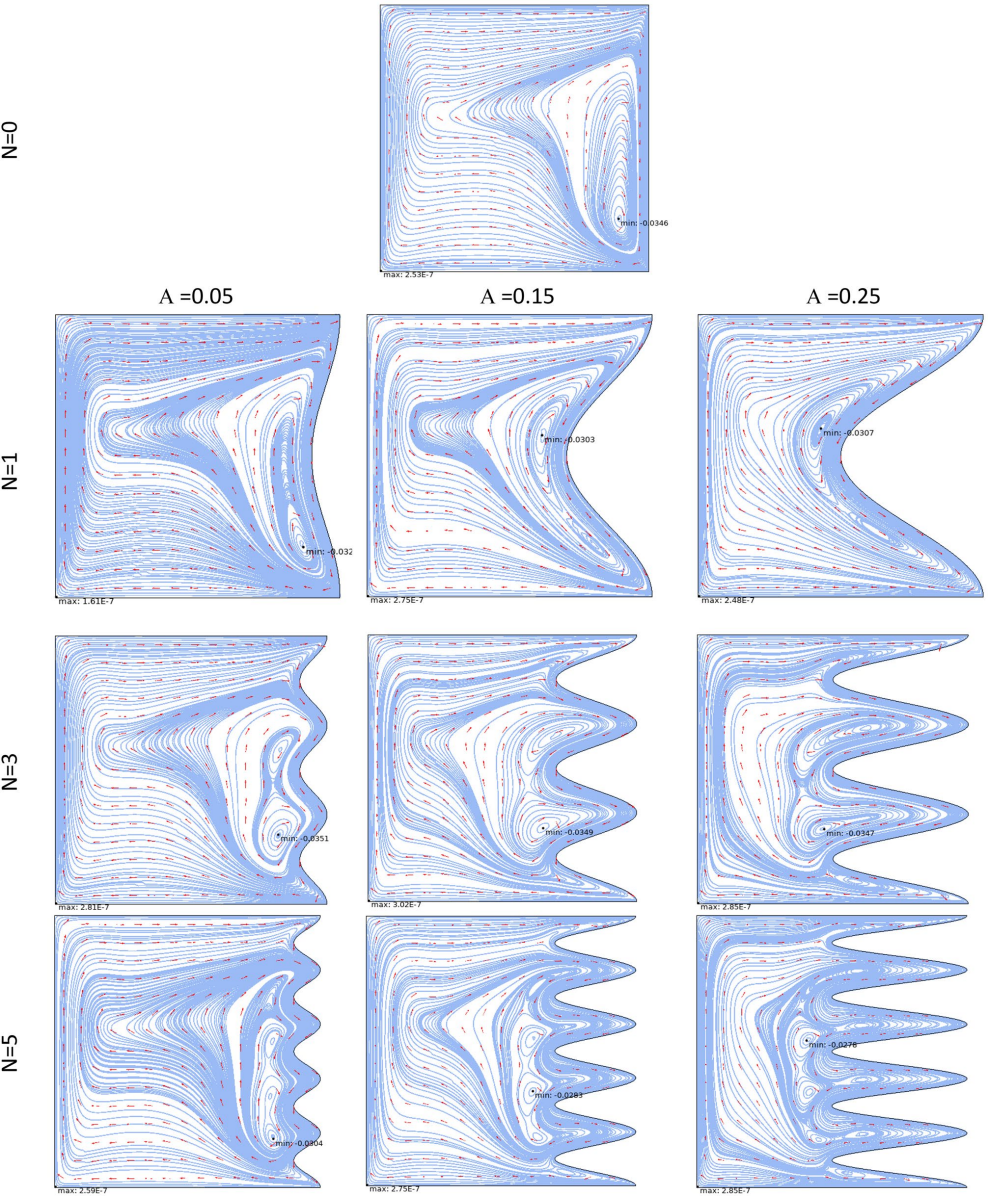
Streamlines and isotherms at (Da = 1e−3, Re = 100, λ = 5, φ = 0.03, Ha = 25, γ = 45, Ri = 10) for Case 1.

**Figure 5:**
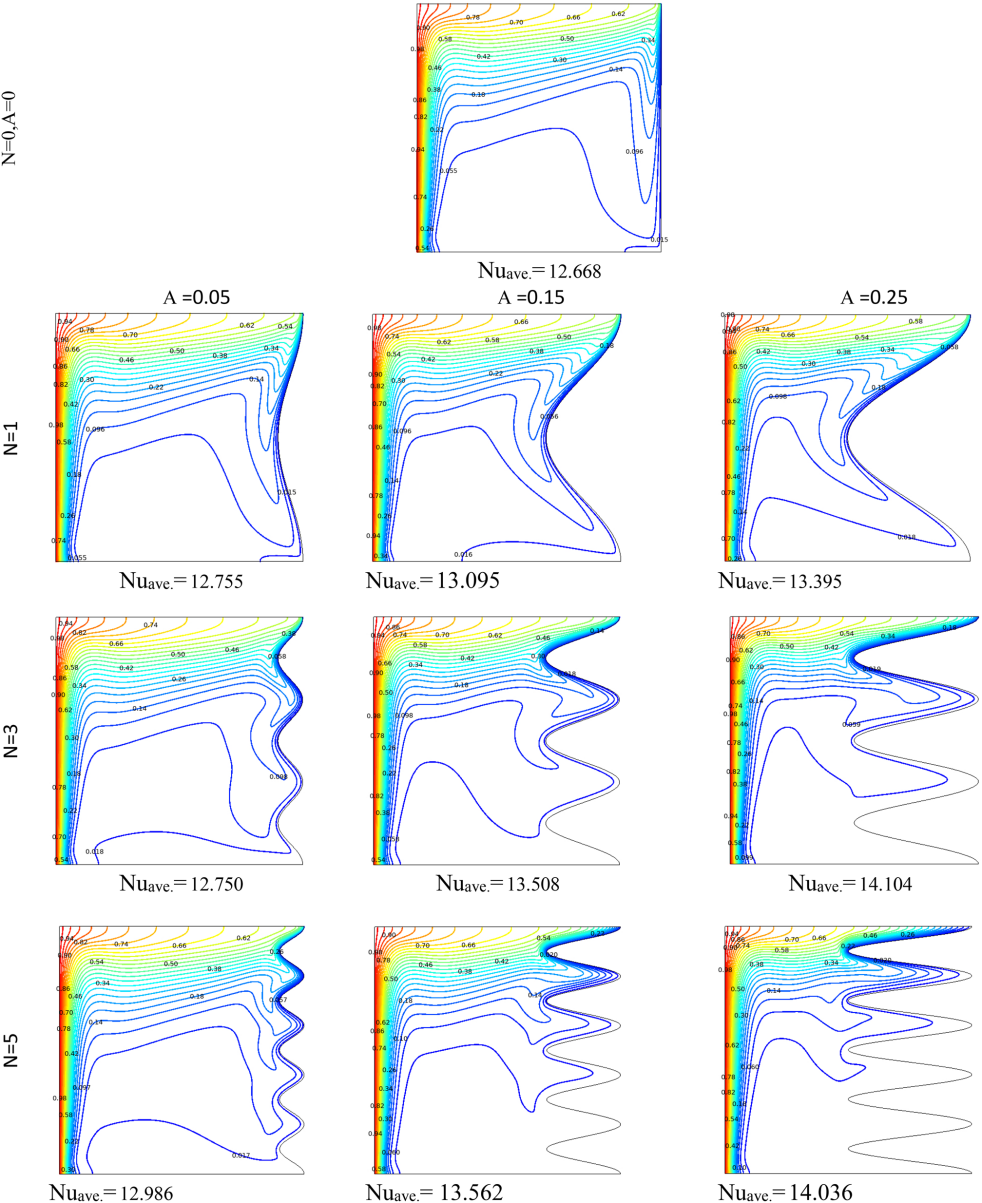
Streamlines and isotherms at (Da = 1e−3, Re = 200, λ = 5, φ = 0.03, Ha = 25, γ = 45, Ri = 10) for Case 1.

**Figure 6:**
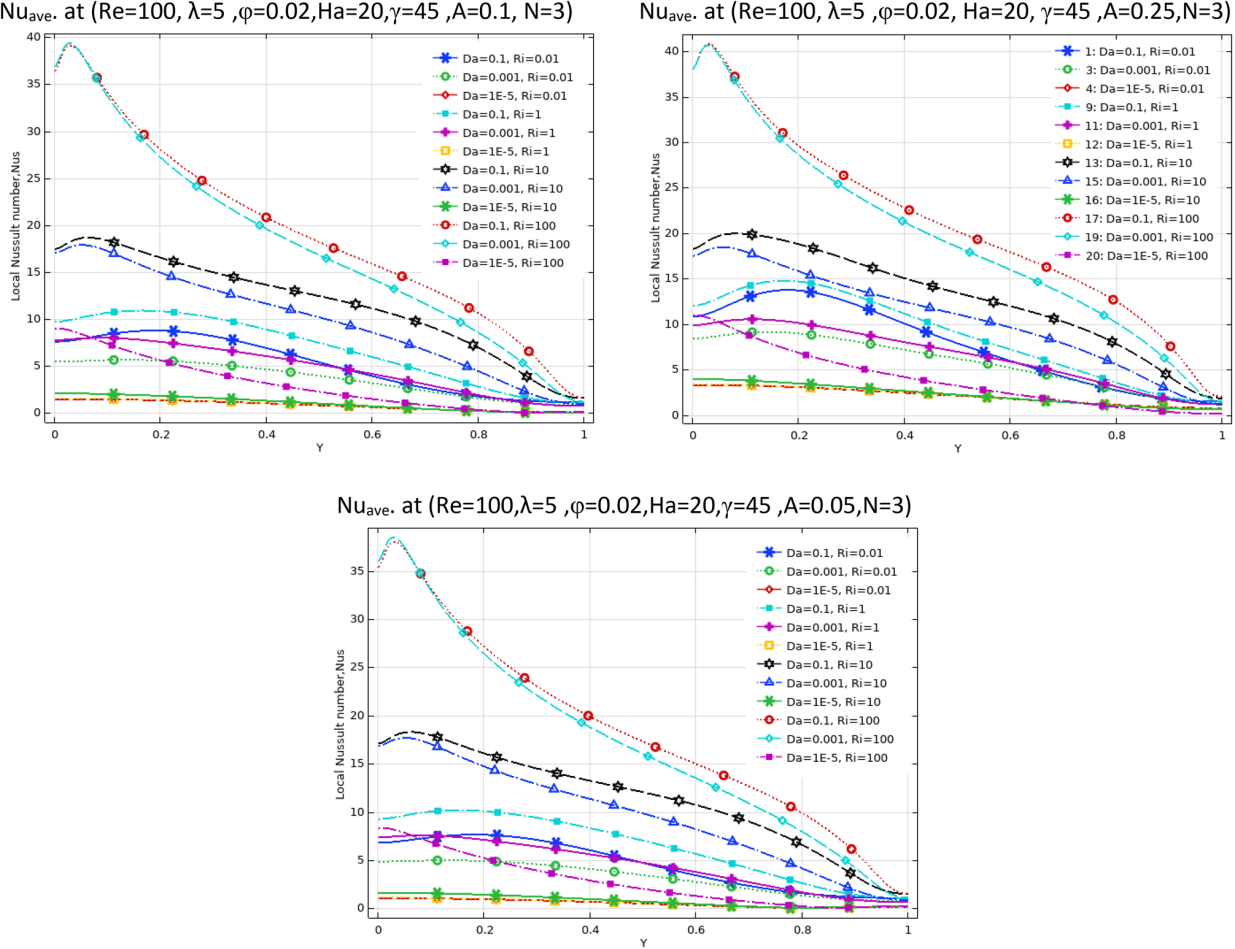
Local Nu number at different Da, Ri and A parameters for Case 1.

**Figure 7:**
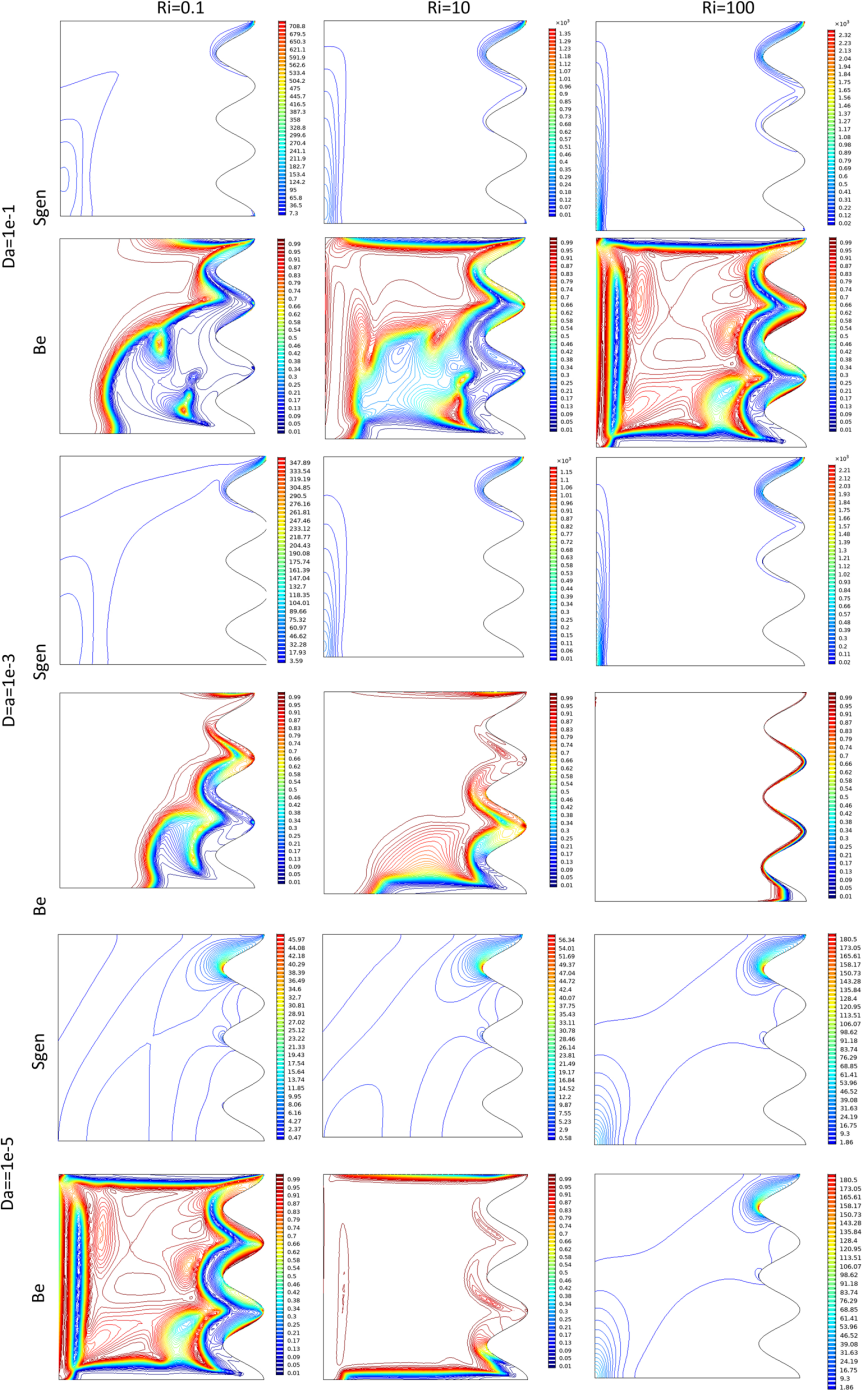
Bejan number and Sgen at different Da and Ri numbers when Re = 100, λ = 5, φ = 0.02, Ha = 20, γ = 45, A = 0.1, N = 3 for case 1.

**Figure 8:**
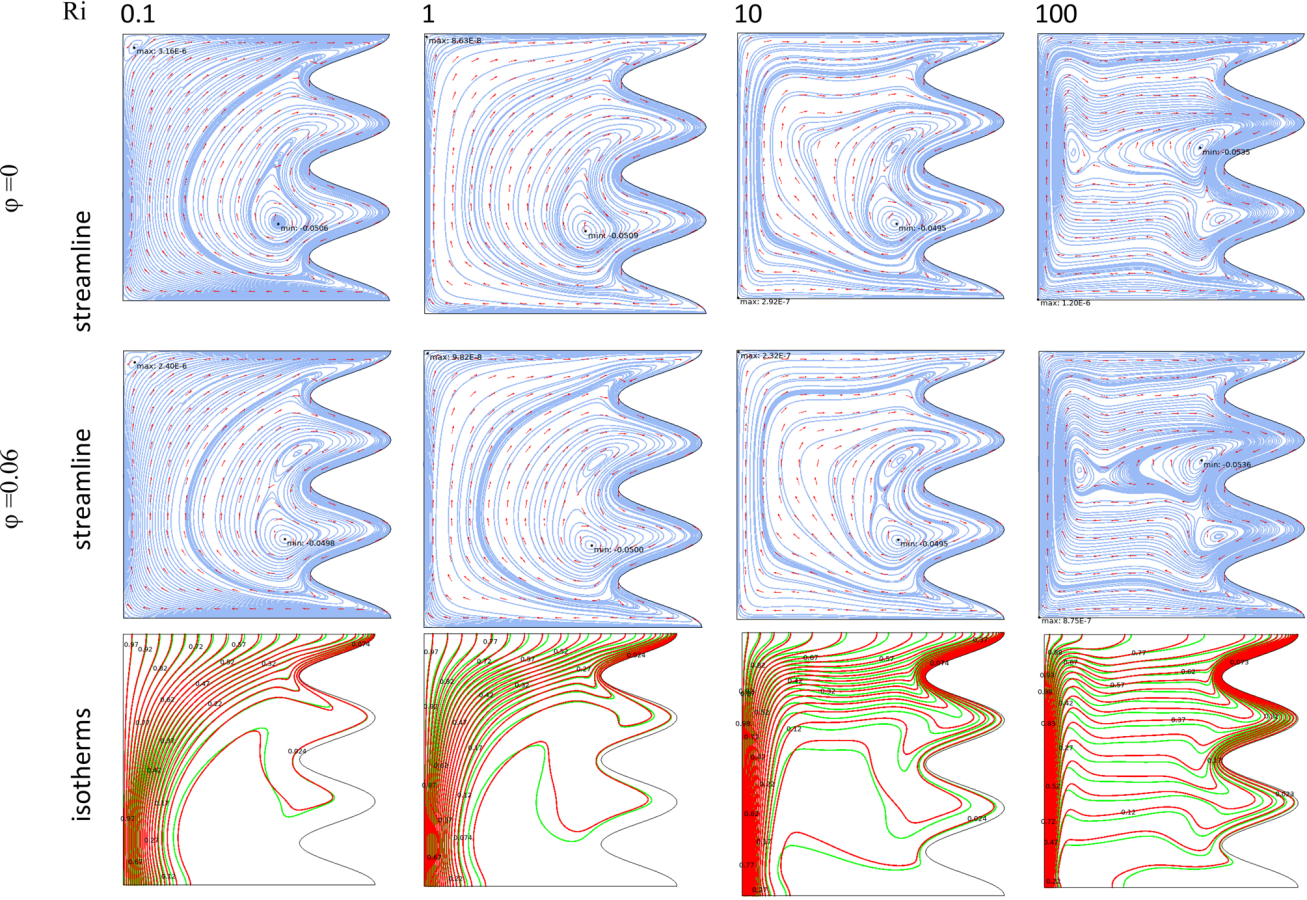
Streamlines an isotherms (Red line at φ = 0.06, green line at φ = 0) when Re = 100, Da = 1e−2, λ = 5, A = 0.15, Ha = 25, γ = 45) for Case 1.

**Figure 9:**
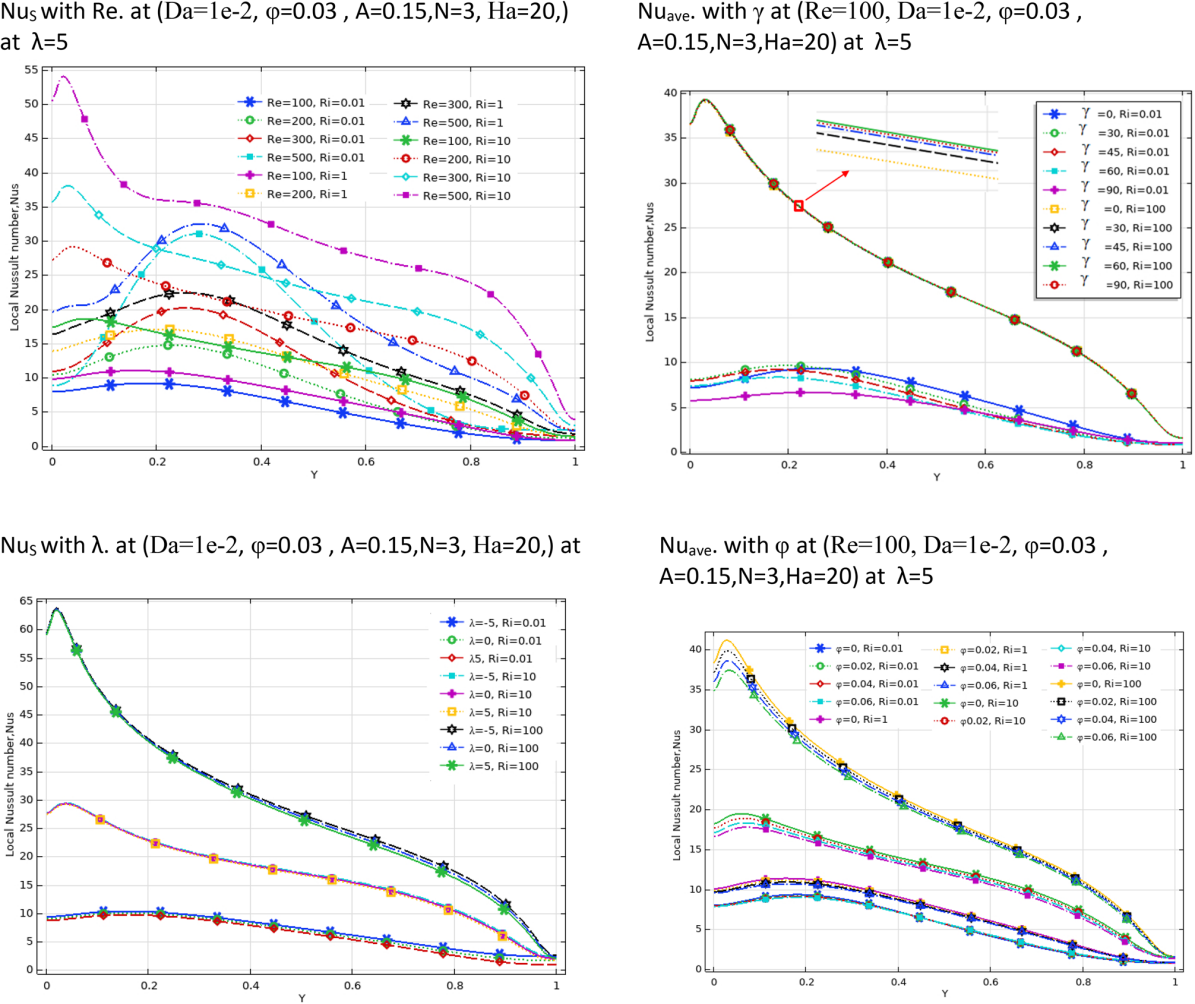
Local Nusselt number for different parameters as variable for Case 1.

**Figure 10:**
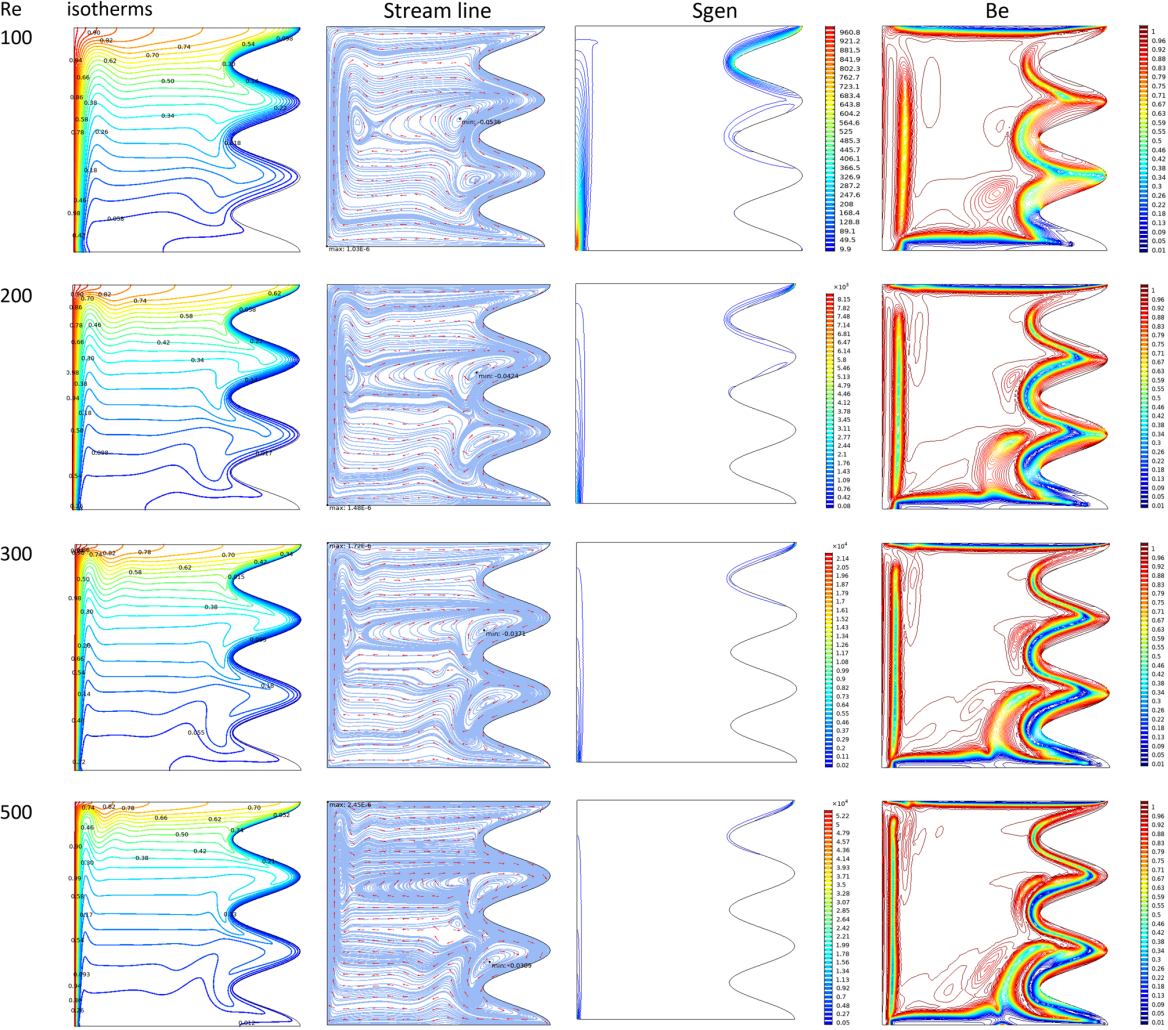
Isotherm, streamlines, Bejan number and Sgen at different Re numbers when Da = 1e−2, A = 0.15, Ha = 20, γ = 45, Ri = 100, γ = 45, φ = 0.03, λ = 5, N = 3 for Case 1.

Figure 3 demonstrates the effect of Da and Ri on the streamlines and isotherms at (Re = 100, λ = 5, φ = 0.02, Ha = 25, γ = 45, A = 0.1, N = 3). As seen, lower Darcy numbers have more uniform temperature and streamlines. Also, for greater Richardson numbers, the flow patterns are more complicated due to natural convection effects in greater numbers, but isotherm lines of the larger Ri numbers are uniform since the forced convection in this condition has minor effect. Figure 4 shows the effect of geometry parameters of wavy wall (A and N) on the streamlines and temperature values when Da = 1e−3, Re = 100, λ = 5, φ = 0.03, Ha = 25, γ = 45, Ri = 10 and Fig. 5 demonstrates the same contour for a greater Re number equal to 200. As seen, Nu_ave_ for the case of square cavity is 12.668 while for all wavy wall its value is increased. It is evident that by increasing the both A and N, the surface of heat transfer is increased and consequently Nu improved in these situations. Table 4 compares these values for better perception on these figures. Tables 5 and 6 compares the results of an average Nusselt number by increasing the Ri and Da numbers at different geometries of wavy wall through changing the A and N parameters. As seen, by increasing the Ri, Nu number is increased significantly due to the natural convection effect, also increasing the Darcy number, improves the Nu number due to more porosity effect on the heat transfer. Figure 6 shows the local Nusselt number of heated wall at different parameters. As seen the maximum local Nusselt numbers occurs for the cases with Ri = 100 and D = 0.1, 0.01 which confirms the discussed issues. Entropy analysis of these cases is presented through Fig. 7 and Table 7 by presenting the different terms of entropy generations and Bejan numbers. To find the effect of nanoparticle volume fraction, Fig. 8 is depicted to see the difference between the two cases. As seen for a greater nanoparticle volume fraction two vortexes are presented in the streamlines which affects the heat transfer as seen in Table 8 and Fig. 9. As seen, increasing the φ has not a significant effect on Nu numbers, but at greater Ri numbers, it reduced the Nu values. Also, Fig. 9 shows the effect of Re, inclined angle and generation parameter in the local Nusselt number. It is observable the Re, γ improves the Nu by their increment, but greater Nu numbers occurs for λ = − 5 compared to λ = 0, 5. Figure 10 investigates the effect of Reynolds number on the streamlines, isotherms and entropy generation parameters.

**Table 4:** Effect of N and A on the average Nusselt number for Case 1.

N	A = 0.05	A = 0.15	A = 0.25
**Nu**_**ave**_** (Da = 1e−3, Re = 200, λ = 5, φ = 0.03, Ha = 25, γ = 45, Ri = 10)case2**			
1	12.755	13.095	13.395
3	12.750	13.508	14.104
5	12.986	13.562	14.036
N = 0, A = 0, Nu_ave_12.668

**Table 5:** Effect of Ri, Da and N on the average Nusselt number for Case 1.

Ri	Da = 1e−1	Da = 1e−2	Da = 1e−3	Da = 1e−5
**Nu**_**ave**_**. at (Re = 100, λ = 5, φ = 0.02, Ha = 20, γ = 45, A = 0.1, N = 3)**				
0.01	4.1957	3.9168	2.9480	0.64390
0.1	4.3486	4.0677	3.0437	0.64540
1	5.5383	5.2574	3.9062	0.66042
10	9.5052	9.3552	7.9813	0.82109
100	15.610	15.518	14.616	2.4287
**Nu**_**ave**_**. at (Re = 100, λ = 5, φ = 0.02, Ha = 20, γ = 45, A = 0.1, N = 1)**				
0.01	3.8612	3.6856	2.9338	0.56271
0.1	4.0285	3.8448	3.0291	0.56427
1	5.2892	5.0743	3.8853	0.58003
10	9.2679	9.1349	7.8634	0.74833
100	14.937	14.851	14.028	2.3216
**Nu**_**ave**_**. at (Re = 100, λ = 5, φ = 0.02, Ha = 20, γ = 45, A = 0.1, N = 5)**				
0.01	3.6856	3.4718	2.7125	0.67002
0.1	3.8739	3.6510	2.8131	0.67149
1	5.2702	5.0171	3.7214	0.68626
10	9.5207	9.3607	7.8982	0.84348
100	15.640	15.547	14.635	2.4462

**Table 6:** Effect of Ri, Da and A on the average Nusselt number for Case 1.

Ri	Da = 1e−1	Da = 1e−2	Da = 1e−3	Da = 1e−5
**Nu**_**ave**_**. at (Re = 100, λ = 5, φ = 0.02, Ha = 20, γ = 45, A = 0.05, N = 3)**				
0.01	3.6765	3.4154	2.5756	0.41216
0.1	3.8566	3. 5916	2.6800	0.41389
1	5.1889	4.9206	3.6122	0.43121
10	9.1969	9.0609	7.7660	0.60950
100	14.944	14.861	14.047	2.2265
**Nu**_**ave**_**. at (Re = 100, λ = 5, φ = 0.02, Ha = 20, γ = 45, A = 0.25, N = 3)**				
0.01	6.3156	6.0406	4.7180	1.6935
0.1	6.3937	6.1182	4.7770	1. 6946
1	7.0916	6.8132	5.3317	1.7059
10	10.476	10.259	8.6754	1.8257
100	17.003	16.890	15.785	3.1776
**Nu**_**ave**_**. at (Re = 100, λ = 5, φ = 0.02, Ha = 20, γ = 45, A = 0.15, N = 3)**				
0.01	4.8147	4.5239	3.4286	0.94378
0.1	4.9405	4.6488	3.5132	0.94517
1	5.9706	5.6792	4.2850	0.95922
10	9.8219	9.6470	8.1954	1.1083
100	16.194	16.091	15.097	2.6579

**Table 7:** Effect of Ri, Da and A on the entropy parameters for Case 1.

Ri	Da = 1e−1			Da = 1e−2			Da = 1e−3			Da = 1e−5		
	SGE	SgeT	Be	SGE	SgeT	Be	SGE	SgeT	Be	SGE	SgeT	Be
**Re = 100, Q = 5, φ = 0.02, Ha = 20, γ = 45, A = 0.25, N = 3**												
0.01	6.7094	6.5883		6.3284	6.3158		5.0285	5.0268		2.2644	2.2642	
0.1	6.7857	6.6647		6.4044	6.3917		5.0864	5.0847		2.2654	2.2653	
1	7.4684	7.3473		7.0850	7.0723		5.6310	5.6293		2.2763	2.2762	
10	10.804	10.682		10.483	10.471		8.9353	8.9335		2.3919	2.3917	
100	17.401	17.255		17.160	17.145		16.072	16.069		3.7304	3.7303	
**at (Re = 100, λ = 5, φ = 0.02, Ha = 20, γ = 45, N = 3, A = 0.15)**												
0.001	5.2421	5.1636		4.8886	4.8803		3.8389	3.8378		1.7737	1.7736	
0.01	5.3642	5.2856		5.0098	5.0016		3.9210	3.9199		1.7749	1.7748	
0.1	6.3640	6.2854		6.0110	6.0027		4.6718	4.6707		1.7872	1.7871	
1	10.168	10.088		9.9285	9.9201		8.5291	8.5280		1.9207	1.9207	
10	16.659	16.558		16.470	16.460		15.500	15.498		3.4132	3.4132	
**at (Re = 100, λ = 5, φ = 0.02, Ha = 20, γ = 45, N = 3, A = 0.05)**												
0.001	4.1412	4.1020		3.8612	3.8570		3.0882	3.0876		1.5434	1.5433	
0.01	4.3148	4.2756		4.0309	4.0267		3.1878	3.1872		1.5445	1.5444	
0.1	5.6016	5.5624		5.3140	5.3097		4.0856	4. 0849		1.5558	1.5558	
1	9.5730	9.5325		9.4076	9.4032		8.1737	8.1730		1.6836	1.6835	
10	15.506	15.447		15.374	15.367		14.584	14.583		3.1728	3.1727

**Table 8:** Effect of Ri, ϕ and λ on the average Nusselt number for Case 1.

Ri	φ = 0	φ = 0.02	φ = 0.04	φ = 0.06
**Nu**_**ave**_**. at (Re = 100, Da = 1e−2, N = 3, A = 0.15, Ha = 20, γ = 45) at λ = 0**				
0.01	4.7668	4.7617	4.7588	4.7583
1	5.9685	5.8663	5.7729	5.6878
10	10.022	9.7933	9.5689	9.3483
100	16.642	16.325	16.015	15.713
**Nu**_**ave**_**. at (Re = 100, Da = 1e−2, N = 3, A = 0.15, Ha = 20, γ = 45)at λ = -5**				
0.01	5.1429	5.1436	5.1467	5.1524
1	6.2621	6.1700	6.0869	6.0123
10	10.253	10.027	9.8049	9.5878
100	17.011	16.688	16.374	16.067
**Nu**_**ave**_**. at (Re = 100, Da = 1e−2, N = 3, A = 0.15, Ha = 20, γ = 45) at λ = 5**				
0.01	4.3726	4.3614	4.3524	4.3454
1	5.6649	5.5522	5.4481	5.3520
10	9.7764	9.5465	9.3199	9.0964
100	16.241	15.929	15.625	15.328

### Case 2

**Figure 11:**
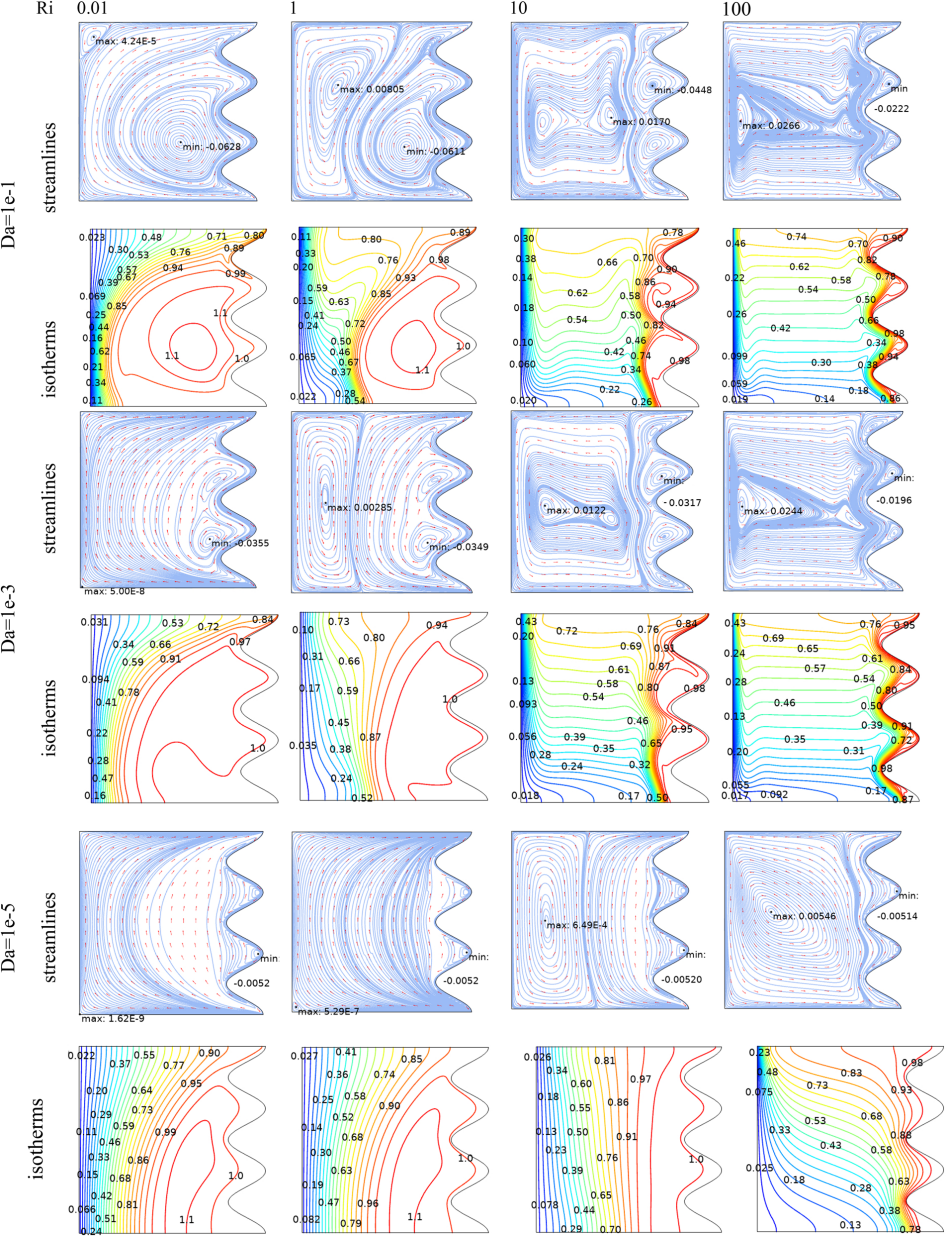
Streamlines and isotherms at (Re = 200, Q = 3, φ = 0.02, Ha = 20, γ = 45, A = 0.1) for Case 2.

**Figure 12:**
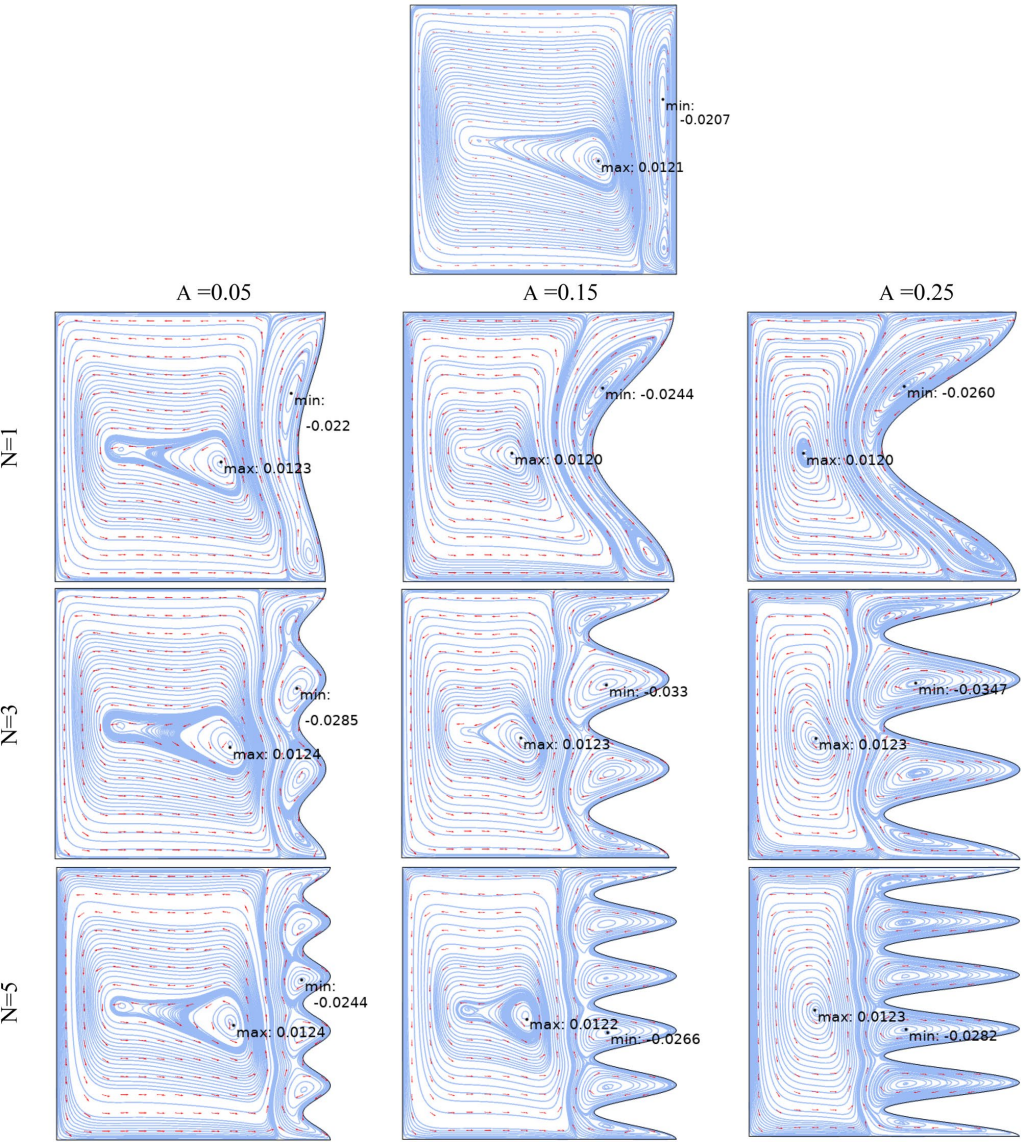
Streamlines and isotherms at (Da = 1e−3, Re = 200, Q = 0, φ = 0.03, Ha = 25, γ = 45, Ri = 10) for Case 2.

**Figure 13:**
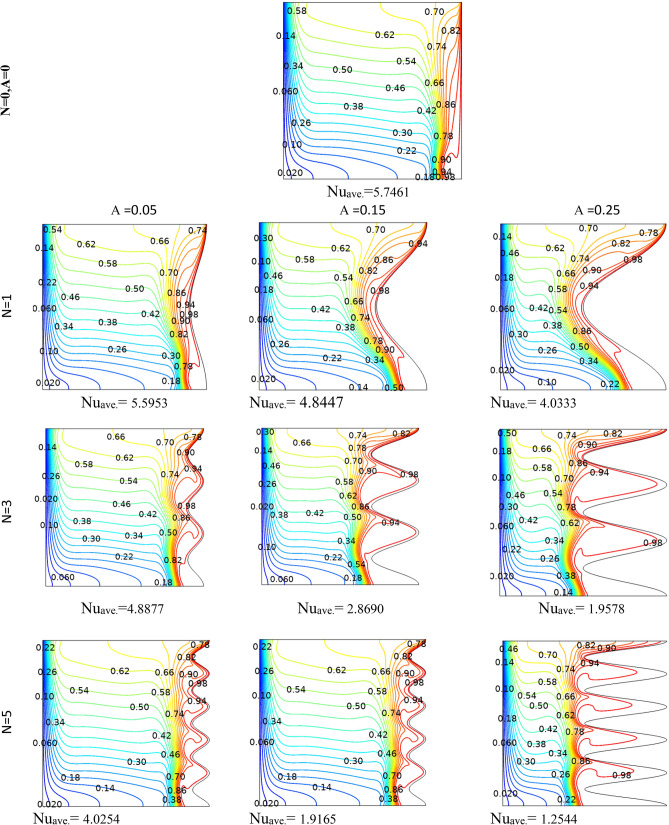
Streamlines and isotherms at (Da = 1e−3, Re = 200, λ = 5, φ = 0.03, Ha = 25, γ = 45, Ri = 10) for Case 2.

**Figure 14:**
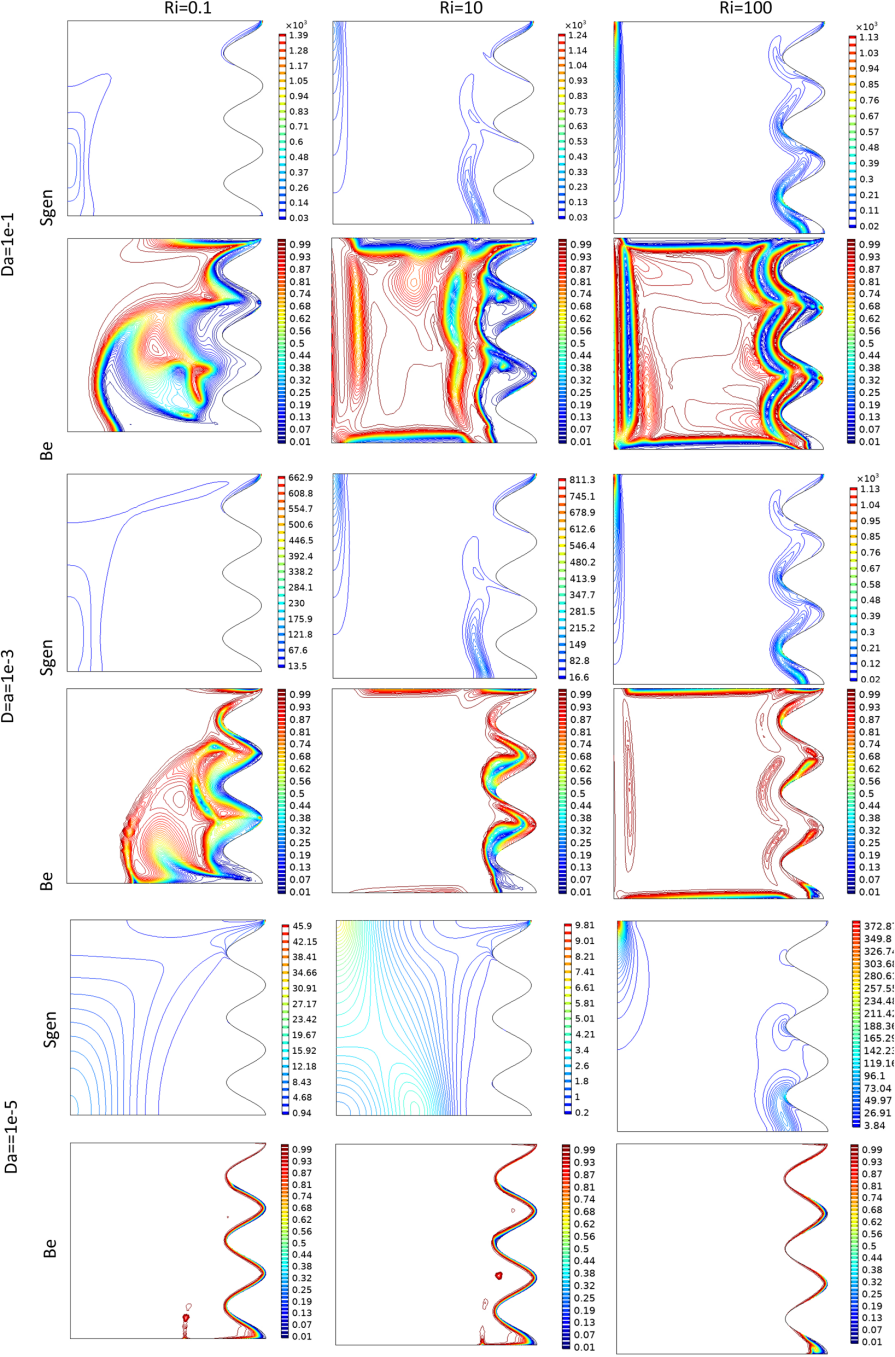
Bejan and Sgen numbers when Re = 200, Q = 3, φ = 0.02, Ha = 20, γ = 45, A = 0.1 for Case 2.

**Figure 15:**
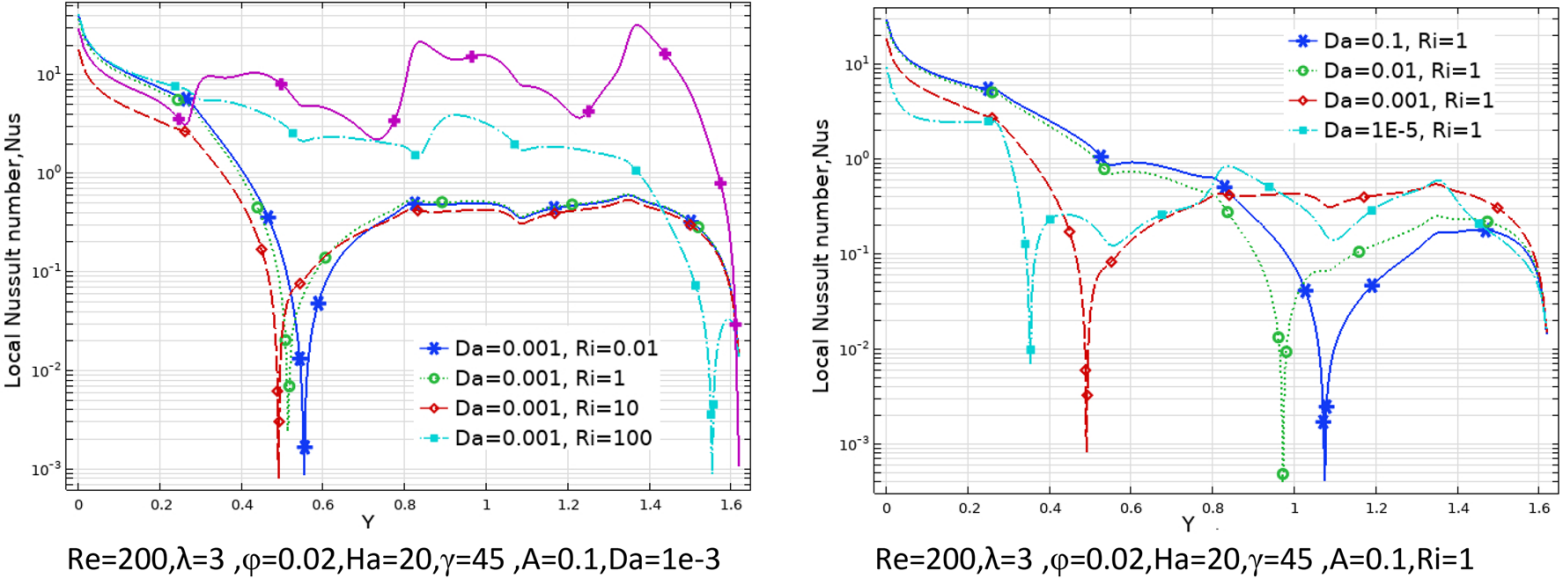
Local Nu numbers along sinusoidal wall at different Ri and Da numbers for Case 2.

By changing the T_h_ and T_c_ positions, case 2 results are obtained and presented here. Table 9 shows the effect of geometry parameters on the average Nusselt number. As seen in this case, all wavy walls have smaller Nu than the square cavity with Nu = 5.7461, and increasing the A and N reduces the Nu number, significantly. Table 10 confirms that in the second case the treatment of Ri and Da increment is the same of Case 1 and improve the Nu number. Figure 11 shows the isotherms and streamlines of second case which is depicted at different Da and Ri numbers. Greater Richardson number causes more uniform isothemlines due to natural convection, while smaller values have more turbulent lines due to more forced convection in heat transfer. Figures 12 and 13 is presented to find the effect of amplitude and number of peaks of wavy wall on the temperatures and streamlines. Table 11 is presented to show the entropy analysis of second case and Table 12 shows the effect of the different nanoparticle volume fraction on the Nu number which have the same treatment of Case 1. Figure 14 is depicted based on the entropy analysis and Bejan number and Sgen numbers are presented at different Ri and Da numbers. Finally, the effect of Ri and Da numbers on the local Nu numbers over the wavy wall is depicted in Fig. 15 for case 2. As seen the greatest values of Nu is related to cases with Da = 0.1 or Ri = 100. To have a comparison between the two tested cases, Tables 13, 14, 15 and 16 are presented. Table 13 compared them at different Ri and λ numbers which confirm the same treatments, but greater values are observed for the Case 1. Table 14 says that increasing the Hartmann number has a negative effect on heat transfer and reduce the Nu values. For the effect of the inclined angle of the magnetic field, Table 15 approves that at low Ri numbers (< 1) increasing the inclined angle, decreases the Nu numbers, but at larger Ri numbers (> 1) it improves the Nu numbers. About the Reynolds number, Table 16 demonstrates that Re number increment can improve the heat transfer of both cases, significantly.

**Table 9:** Effect of N and A on the average Nusselt number for Case 2.

N	A = 0.05	A = 0.15	A = 0.25
**Nu**_**ave**_** (Da = 1e−3, Re = 200, λ = 5, φ = 0.03, Ha = 25, γ = 45, Ri = 10), case2**			
1	5.5953	4.8447	4.0333
3	4.8877	2.8690	1.9578
5	4.0254	1.9165	1.2544
N = 0, A = 0, Nu_ave=_5.7461

**Table 10:** Effect of Ri, Da and A on the average Nusselt number for Case 2.

Ri	Da = 1e−1	Da = 1e−2	Da = 1e−3	Da = 1e−5
**Nu**_**ave**_**. at (Re = 100, Q = 5, φ = 0.02, Ha = 20, γ = 45, A = 0.1, N = 3) case 2**				
0.01	1.5467	1.4898	1.1890	0.53485
0.1	1.4752	1.4222	1.1501	0.53555
1	0.82741	0.79796	0.78426	0.54235
10	1.9424	1.8524	1.2103	0.58679
100	4.8145	4.7652	4.3395	0.41226
**Nu**_**ave**_**. at (Re = 100, Q = 5, φ = 0.02, Ha = 20, γ = 45, A = 0.05, N = 3) case 2**				
0.01	1.6341	1.5639	1.1995	1.0166
0.1	1.5298	1.4678	1.1523	1.0175
1	0.82833	0.80437	0.89064	1.0261
10	2.6197	2.4952	1.6092	1.0667
100	6.3329	6.2715	5.7379	0.46306
**Nu**_**ave**_**. at (Re = 100, Q = 5, φ = 0.02, Ha = 20, γ = 45, A = 0.25, N = 3) case 2**				
0.01	1.4767	1.4333	1.2508	0.50949
0.1	1.4547	1.4120	1.2352	0.50907
1	1.2208	1.1857	1.0690	0.50483
10	1.0046	0.95384	0.68675	0.46381
100	2.4689	2.4425	2.2167	0.46094
**Nu**_**ave**_**. at (Re = 100, Q = 5, φ = 0.02, Ha = 20, γ = 45, A = 0.15, N = 3) case 2**				
0.01	1.4780	1.4278	1.1872	0.44121
0.1	1.4299	1.3820	1.1581	0.44063
1	0.93636	0.89045	0.85694	0.43491
10	1.4907	1.4216	0.93396	0.38476
100	3.7092	3.6702	3.3356	0.40811

**Table 11:** Effect of Ri, Da on the entropy parameters for Case 2.

Ri	Da = 1e−1			Da = 1e−2			Da = 1e−3			Da = 1e−5		
	SGE	SgeT	Be	SGE	SgeT	Be	SGE	SgeT	Be	SGE	SgeT	Be
**Re = 200, Q = 3, φ = 0.02, Ha = 20, γ = 45, A = 0.1, case2**												
**0.01**	6.7878	6.7274	0.9911	6.2318	6.2255	0.999	4.6444	4.6436	0.9998	2.0694	2.0694	1
**0.1**	6.4436	6.3832	0.9906	5.8669	5.8605	0.9989	4.3530	4.3521	0.99979	2.0626	2.0625	.99995
**1**	4.5030	4.4422	0.9806	4.1672	4.1608	0.99846	2.7421	2.7412	0.99967	1.9959	1. 9959	1
**10**	7.5480	7.4827	0.9913	7.3516	7.3448	0.999	6.2162	6.2152	0.99984	1.5983	1.5983	1
100	13.596	13.451	0.9893	13.401	13.386	0.9988	12.795	12.793	0.999843	3.8534	3.8534	1

**Table 12:** Effect of Ri and ϕ on the average Nu for Case 2.

Ri	φ = 0	φ = 0.02	φ = 0.04	φ = 0.06
**Nu**_**ave**_**. at (Re = 100, Da = 1e−2, N = 3 , A = 0.15, Ha = 20, γ = 45) at λ = 5, case 2**				
0.01	1.4402	1.4278	1.4158	1.4045
1	0.85150	0.89045	0.92915	0.96704
10	1.4954	1.4216	1.3498	1.2801
100	3.8172	3.6702	3.5322	3.4024

**Table 13:** Comparsion of average Nu for Case 1 and Case 2 at different Ri and λ.

Ri	λ = − 5	λ = 0	λ = 5
**Nu**_**ave**_**. at (Re = 200, Da = 1e−3, φ = 0.02, A = 0.15, Ha = 25, γ = 45), N = 3, case 1**			
0.01	5.6066	5.2690	4.9205
1	7.4195	7.1768	6.9296
10	13.671	13.508	13.340
100	23.762	23.447	23.112
**Nu**_**ave**_**. at (Re = 200, Da = 1e−3, φ = 0.02, A = 0.15, Ha = 25, γ = 45), N = 3, case 2**			
0.01	3.7794	2.4500	1.8625
1	2.6110	1.3690	0.93481
10	3.5444	2.8690	2.1541
100	6.5596	6.0531	5.5125

**Table 14:** Comparison of average Nu for Case 1 and Case 2 at different Ri and Ha.

Ri	H = 0	H = 20	H = 40	H = 60
**Nu**_**ave**_**. at (Re = 100, Da = 1e−2, φ = 0.03, A = 0.15, N = 3, γ = 45) at λ = 5, case 1**				
0.01	5.1323	4.3568	3.5438	3.0553
1	6.2024	5.4991	4.5194	3.7893
10	9.7804	9.4328	8.5256	7.4314
100	15.996	15.776	15.144	14.216
**Nu**_**ave**_**. at (Re = 100, Da = 1e−2, φ = 0.03, A = 0.15, N = 3, γ = 45) at λ = 5, case 2**				
0.01	1.5995	1.4218	1.1967	1.0623
1	1.0244	0.90986	0.84349	0.81745
10	1.5397	1.3855	1.0443	0.72009
100	3.6809	3.6001	3.3853	3.0935

**Table 15:** Comparison of average Nu for Case 1 and Case 2 at different Ri and γ.

Ri	γ = 0	γ = 30	γ = 45	γ = 60	γ = 90
**Nu**_**ave**_**. at (Re = 100, Da = 1e−2, φ = 0.03, A = 0.15, N = 3, H = 20, ) at λ = 5, case 1**					
0.01	4.8557	4.6335	4.3567	4.0319	3.6453
1	5.7758	5.6569	5.4991	5.3187	5.0991
10	9.3907	9.4205	9.4328	9.4413	9.4376
100	15.759	15.767	15.776	15.786	15.796
**Nu**_**ave**_**. at (Re = 100, Da = 1e−2, φ = 0.03, A = 0.15, N = 3, H = 20, ) at λ = 5, case 2**					
0.01	1.5835	1.5034	1.4218	1.3359	1.2546
1	1.1742	1.0356	0.90986	0.78448	0.67669
10	1.3104	1.3456	1.3855	1.4263	1.4697
100	3.5729	3.5871	3.6001	3.6126	3.6234

**Table 16:** Comparison of average Nu for Case 1 and Case 2 at different Ri and Re.

Ri	Re = 100	Re = 200	Re = 300	Re = 500
**Nu**_**ave**_**. at (, Da = 1e−2, φ = 0.03, A = 0.15, N = 3, H = 20, ) at λ = 5, case 1**				
0.01	4.3567	6.7333	8.7500	12.018
0.1	4.4804	6.9869	9.0991	12.453
1	5.4991	8.8444	11.539	15.723
10	9.4328	14.534	18.406	24.433
100	15.776	23.875	30.070	39.877
**Nu**_**ave**_**. at (, Da = 1e−2, φ = 0.03, A = 0.15, N = 3, H = 20, ) at λ = 5, case 2**				
0.01	1.4218	2.3380	3.0636	4.2430
0.1	1.3786	2.2295	2.9070	4.0775
1	0.90986	1.2082	1.6113	2.5463
10	1.3855	2.6788	3.6929	5.3592
100	3.6001	5.8179	7.6061	10.534

## Conclusion

In this paper, GFEM was used for modeling the CNT-water nanofluids in a wavy porous cavity with movable and different boundary conditions under the magnetic field. Richardson (Ri), Darcy (Da), Hartmann angle (γ), Amplitude (A), Number of peaks (N), Volume fraction (φ), Heat generation factor (λ), Hartmann number (Ha) and Reynolds number (Re) effects were studied on the results of entropy generation and Nu numbers. Results indicated that by increasing the Ri, Nu number is increased significantly due to the natural convection effect, also increasing the Darcy number, improves the Nu number due to more porosity effect on the heat transfer. The presence of the wavy wall increases the Nusselt number, thus it reinforces heat transfer, this reinforcement, increasing with the increase of both number of undulation and its amplitude. Also, greatest values of Nu are occurring for the case with Da = 0.1 or Ri = 100.

## List of symbols

A: Amplitude (m)
B^*^: External Magnetic field
B_o_: Magnetic field strength (T)
Be: Bejan number
C_p_: Specific heat at constant pressure (KJ/kg K)
Da: Darcy number
g: Gravitational acceleration (m/s^2^)
Ha: Hartman number
k: Thermal conductivity (W/m K)
*K*: Permeability of porous medium (m^2^)
L: Length and height of the cavity (m)
N: Number of peaks
*Nu*_*s*_: Local Nusselt number
Nu_ave_: Average Nusselt number
*p*: Pressure (Pa)
P: Dimensionless pressure ($$\frac{p}{{\rho }_{nf}{V}_{o}^{2}}$$)
Pr: Prandtl number (ν_bf_/α_bf_)
Q: Heat generation source (W/m^2^)
Ra: Rayleigh number $(g{\beta }_{bf}{L}^{3}({T}_{h}-{T}_{\mathrm{c}})/{\nu }_{bf}{\alpha }_{bf})$
Ri: Richardson number (Ri = Gr/Re^2^)
S: Entropy generation (W/K m^3^)
S_1_, S_T_: Dimensionless partial slip parameters
*T*: Temperature (K)
T_c_: Wall with cold temperature
T_h_: Wall with hot temperature
T_o_: Reference Temperature
u, v: Components velocity in Cartesian coordinates (m/s)
$$\nabla u$$: Velocity vector
$U, V$: Dimensionless velocity component
${V}_{o}$: Velocity of lid-driven (m/s)
X, Y: Dimensionless coordinate
*x, y*: Cartesian coordinates (m

## Greek symbols

α: Thermal diffusivity (m^2^/s)
$\beta$: Thermal Expansion coefficient (1/K)
$$\theta $$: Dimensionless temperature (T − T_c_/T_h_ − T_c_)
$\lambda$: Heat generation factor
$\Psi$: Dimensionless stream function
μ: Dynamic viscosity (kg s/m)
$$\sigma $$: Electrical conductivity
ν: Kinematic viscosity (μ/ρ)(Pa s)
γ: Hartman angle
φ: Nanoparticle volume fraction (%)
$$\Theta $$: Irreversibility factor
$$\varnothing $$: Basic function
Ω: Electrical potential
ρ: Density (kg/m^3^

## Subscripts

c: Cold
*f*: Base Fluid (pure)
sp: Solid particle
*nf*: Nanofluid
so: Conductive solid cylinder
h: Hot

## Abbreviations

Cond.: Conduction
GFEM: Galerkin finite elements method
Conv.: Convection
CNT: Carbon nano-tube
